# The Causal Inference of Cortical Neural Networks during Music Improvisations

**DOI:** 10.1371/journal.pone.0112776

**Published:** 2014-12-09

**Authors:** Xiaogeng Wan, Björn Crüts, Henrik Jeldtoft Jensen

**Affiliations:** 1 Department of Mathematics and Centre for Complexity Science, Imperial College London, London, United Kingdom; 2 Brainmarker BV, Molenweg 15a, Gulpen, The Netherlands; University of L′Aquila, Italy

## Abstract

We present an EEG study of two music improvisation experiments. Professional musicians with high level of improvisation skills were asked to perform music either according to notes (composed music) or in improvisation. Each piece of music was performed in two different modes: strict mode and “let-go” mode. Synchronized EEG data was measured from both musicians and listeners. We used one of the most reliable causality measures: conditional Mutual Information from Mixed Embedding (MIME), to analyze directed correlations between different EEG channels, which was combined with network theory to construct both intra-brain and cross-brain networks. Differences were identified in intra-brain neural networks between composed music and improvisation and between strict mode and “let-go” mode. Particular brain regions such as frontal, parietal and temporal regions were found to play a key role in differentiating the brain activities between different playing conditions. By comparing the level of degree centralities in intra-brain neural networks, we found a difference between the response of musicians and the listeners when comparing the different playing conditions.

## Introduction

Improvisation, an instantaneous creative behavior, is often encountered in different forms of art such as music and dance. In this paper, we study the brain mechanisms of music improvisation. We refer to the performance according to notes as composed music, while the instantaneous creative performance as improvisation. In the performances, each piece of music either composed or improvised was played in either a mechanical manner (i.e. strict mode) or in a more emotionally rich manner (“let-go” mode).

Music improvisation is believed to involve neural-substrates in large brain regions [16] [18] [19] [6] [4] [3] [2] [1] [12] [13]. If these large brain regions are identified, one could use neuro-scientific approaches to improve the quality of music performance. To analyze the brain mechanisms of music improvisation, we use causality measures to analyze the EEG data measured from the experiments and construct generalized neural networks for each experimental condition. We have investigated various kinds of causality measures and found that the conditional Mutual Information from Mixed Embedding (MIME, a time domain direct causality measure developed by I. Vlachos and D. Kugiumtzis [27]) to be the optimal causality measure for our EEG analysis. In this paper, we present our results on intra-brain and cross-brain neural networks, and compare the networks observed for different playing conditions.

Music improvisation has long been studied by neuroscientists and mathematicians using various approaches. Recent research has identified a number of frontal brain regions, including the pre-supplementary motor area (pre-SMA) [7] [18], [22] and the dorsal premotor cortex (PMD) [9] [15], to play central roles in more cognitive aspects of movement sequencing and creative generation of music. For a while, scientists have used brain scanning techniques such as fMRI, PET and EEG to study the brain. O. D. Manzano et. al. used fMRI to study the melodic and rhythmic improvisation in a 2×2 factorial experiment [18] [19], where the dorsal premotor cortex (PMD, in frontal cortex, is assumed to be consistently involved in cognitive aspects of planning and selection of spatial motor sequences) was found to be the main region for melodic improvisation, while the pre-supplementary motor area (pre-SMA, showing increased activation during perception, learning and reproduction of temporal sequences) was identified to be related to rhythmic improvisation [18] [19]. A. L. Berkowitz et. al. [3] also used fMRI in a study of expertise-related neural differences between musicians and non-musicians during improvisation. Their results show that musicians have right temporoparietal junction (rTPJ) deactivation during music improvisation, while non-musicians showed no activity change in this region [3]. Moreover, C. Babiloni et al. [1] studied the frequency filtered EEG measured from professional saxophonists during music performances, they found the EEG power density values decreased in the alpha band (8–12 Hz) in the posterior cortex during resting state, while the power values enhanced within narrow high-frequency bands during music performances [1]. Other studies are e.g. EEG phase synchrony analysis [5], fMRI study of jazz improvisation [16], PET studies of melody and sentence generation [6] and the fMRI study of pseudo-random motor and cognitive tasks [2].

Amongst the many mathematical tools used to study music improvisation, we consider the most relevant tools to be measures of correlations, standardized Low Resolution Brain Electromagnetic Tomography (sLORETA, [21]) and analysis of variance (ANOVA, [20], [23]). sLORETA is a method used to localize, identify and visualize EEG point sources in the brain [21]. ANOVA is used to analyze the statistical differences between different experimental conditions [20] [23]. It has been applied to e.g. the music improvisation study on trained pianists [4], which revealed that the dorsal premotor cortex, the rostral cingulate zone of the anterior cingulate cortex and the inferior frontal gyrus are important to both rhythmic and melodic motor sequence creation [4]. D. Dolan et al. undertook a sLORETA analysis of the EEG for music improvisation [10]. They used part of the same data as we have done for the analysis described in this paper, namely EEG measured on members of a trio and two members of the audience. Their sLORETA analysis suggested similar results to those we obtained from the MIME analysis (see below). In both cases the frontal cortex was found to be strongly involved in music improvisation [10]. Other studies investigated e.g. the correlation analysis of EEG [11] [14] and music performance [12] [13], but these investigations did not address the *direction* of causal influence between EEG channels. In the present paper, we will complement the analysis in [10] by applying MIME and network theory to the analysis of the multi-channel EEG data streams.

For our EEG analysis, we have investigated three popular measures, namely the nonlinear indirect measures: transfer entropy (TE [24]) and MIME [27], and a linear direct measure: partial directed coherence (PDC [25] [26]). TE was found to unsatisfactorily resolve the direction of the causal flow, while PDC returns false or unreliable causalities presumably due to the limitations of the linear autoregressive model fitting. MIME appears to be the best measure for our EEG analysis, among the measures we investigated, and is able to efficiently generate reliable and robust causality results with a satisfactory resolution. Hence, we only report the MIME analysis of the neural intra-brain and inter-brain information flow between large brains regions of musicians and listeners. The technical details of the comparison between different causality measures will be discussed in another publication.

## Our study

We consider two experiments regarding the effects of the performance approach for different types of music (composed music and improvisation) and different playing modes (strict mode and “let-go” mode). Our main interest is to identify neural information flow between EEG channels using MIME. It is noteworthy that our emphasis was put on making the experimental environment as close as possible to real concert performances. This emphasis was especially addressed in the second experiment, whose playing environment is more extensive.

Two music improvisation experiments were done at the Guild Hall School of Music and Drama in London separately on 20.06.2010 and 31.03.2012. Synchronized EEG measurements from both the musicians and the listeners were collected by Björn Crüts and his team (BrainMarker Corp.) using CE-certified EEG device (Brainmarker, the Netherlands) during the music performances.

In the first experiment the international concert pianist David Dolan solo performed four pieces of music:

**Test 1:** Schubert-Impromptu in G flat major Op. 90 No. 3, neutral mode, uninvolved

**Test 2:** Schubert-Impromptu in G flat major Op. 90 No. 3, fully involved

**Test 3:** Improvisation, polyphonic, intellectual exercise

**Test 4:** Improvisation, polyphonic, emotional letting go.

The audience consisted of one listener. Both participants were connected to synchronized EEG amplifiers (250 Hz sampling frequency) with 8 electrodes (P4, T8, C4, F4, F3, C3, T7, P3). The electrodes are labeled by the initial of the corresponding cortices, P: parietal cortex (perception, multi-sensory integration), T: temporal cortex (processing of language and sounds), C: central cortex (sensory and motor function), F: frontal cortex (attention and executive control). The odd numbers stand for locations on the left brain, while even numbers represent locations on the right brain. The electrodes are all localized according to the international 10–20 system (Jasper, 1958). A reference electrode (Cz) at the central location on the top of the head was used, so that each EEG signal was mono-polar referenced to this central site and activity levels of the eight sites could be compared relative to each other.

In the second experiment, the music was performed by the Trio Anima (three highly acclaimed musicians: Drew Balch (violist), Matthew Featherstone (flutist) and Anneke Hodnett (harpist)) in the following order:

- Ibert [duration: 3′ 30′′]: 1. strict & 2. “let-go”
- Telemann [duration: 2′]: 1. “let-go” & 2. strict
- Improvisation: 1. “let-go” & 2. strict
- Ravel [duration: 2′ 50′′]: 1. strict & 2. “let-go”
- Improvisation: 1. strict & 2. “let-go”

The audience consisted of 14 listeners. Synchronized EEG data was measured from all the musicians and from only two of the listeners, this was due to technical limitations on the number of EEG machines. However, the data from one listener had to be excluded from the cross-brain analysis, due to a technical issue concerning the synchronization with the other EEG machines. The EEG data (100 Hz sampling frequency) was measured from 10 electrodes: P4, T8, C4, F4, F3, C3, T7, P3, O1 and O2 (O: occipital cortex, visual processing center).

In this experiment, pieces A, B and D are composed music (music performances according to a written score) and as such are similar to the first two tests pieces in the first experiment. Pieces C and E were entirely improvised (instantaneous creative performance of music) by the trio and therefore similar in performance approach to the last two pieces of the first experiment. Both the composed music and the improvised were played in the strict mode and the “let-go” mode. Similar to the mode played in the test 2 and test 4 of the first experiment, the “let-go” is a music playing mode with full emotional expression, whereas the strict mode is a mechanical rendition of music, which is similar to the neutral mode in test 1 of the first experiment. However, the intellectual exercise (test 3) of the first experiment consists of the musician improvising a technically correct piece of music without any emotional content. This mode wasn't used in the second experiment. Our aim is to identify the neural differences between the different modes of performance.

For the EEG measurements, standard EEG caps (BraiNet, Jordan Neuroscience) were used to standardize the electrode locations by using anatomical reference points. This ensures that measurements within and between subjects could be compared. Ag/AgCl electrodes with carbon shielded wires (Temec, the Netherlands) and conductive electrode gel (Ten20, D.O. Weaver & Co) were used to minimize movement artifacts. Data acquisition was carried out with a sample frequency of 250 Hz in the first experiment and 100 Hz in the second experiment. Data filtering was executed using a first order 0.16 Hz high pass filter and 59 Hz fourth order low pass filter. The amplifiers were time-synchronized using a purpose build external trigger. Before the measurement, the skin was cleaned using abrasive gel (NuPrep, D.O. Weaver & Co.) to ensure low skin impedance (5 k Ω) and high signal quality.

The reason for the use of 8 channel EEG recordings, is that we focus on the activities of large cortical brain regions, such as the primary motor or temporal cortex. In addition, we aim to choose an experimental set-up of minimal discomfort for the musicians, but still apply enough electrodes to distinguish the activity from different large brain regions. Similar approaches have been used in other patient studies e.g. studies of autism, where the motor cortex activity was measured. Due to the machine set-up (active shielding mechanism), movement artifacts were minimized. Since prefrontal poles were not measured, eye movement artifacts were excluded. Similar considerations hold for other muscle activity, which most frequently originate in the prefrontal cortex, this region was excluded from our measurement. Concerning muscle activity originating in the temporal regions (T), we note that that such activity is limited to high frequencies and that these frequency components (>32 Hz) were filtered out via Fourier transforms.

According to Dr. David Dolan, the redering of composed and improvised music in our experiments are mainly distinguished by the overall manner of the music performances [10]. Improvisation contains more coherent and long-term structural lines, shared by all members of the ensemble. The short-term beats are freer and uneven, but the deep, longer-term pulse is extremely stable in improvisation. In performances of composed music, the gestures seemed to be shorter and more rigid (even in quick repetitive phrases). There is less room for spontaneity and the audience finds themselves less surprised. This is perhaps the reason behind the results of psychological tests, which showed that the audiences found improvisation to be more emotionally engaging and musically interesting [10]. Extra notes were added spontaneously by the freer distribution of time over gestures, which leans more significantly on structural key moments. Another important characteristic of improvisation, is that the risk-taking and mutual support are provided spontaneously by the members of the ensembles. This is probably a consequence of the higher level of active listening that is needed during the improvisation. Hence, one may expect that when improvising, musicians are prevented from entering into an ‘autopilot mode’ since the improvisation forces them to listen very attentively to the music while being prepared for the unexpected to happen at any instance.

Previous research has identified a number of the frontal regions, including the pre-supplementary motor area (pre-SMA) [7] [18] [22] and the dorsal premotor cortex (PMD) [9] [15] to play central roles in more cognitive aspects of the movement sequencing and creative generation of music. We hypothesize that the musicians may trigger more wide-distributed neural networks when improvising than when performing composed music, and that the frontal regions (attention and executive control) play an important role in the improvisation process.

Given the above differences between different music types and playing modes, we aim to investigate their neural substrates based on the network structure of the neural information flow. Previous music improvisation studies considered either unique point sources of the EEG [10] or symmetric correlations between brain regions [7] [22] [9] [15]. In this paper, we present a causality analysis of the EEG data recorded during music improvisation, which aims to identify neural differences between experimental conditions. We use the MIME causality measure to analyze the EEG data, and construct both intra-brain and cross-brain networks from the MIME causalities. We mention one limitation of MIME. Namely that since MIME is a bivariate causality measure (in contrast to multivariate measures) it is unable to distinguish between direct and indirect causalities. (By this we mean the following. Consider three time series 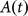, 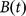 and 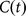. Assume 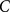 depends on 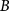 and 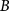 depends on 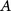, but that 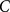 doesn't depend directly on 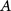. MIME would however return the following dependencies: 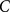 on 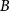 and 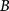 on 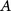 and also 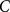 on 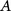, because it is a bivariate measure, it is unable to determine that the dependence of 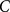 on 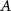 is only through 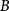. We stress that although MIME is not a direct measure, in the sense just mentioned, it is certainly a *directed* measure, in the sense that it can determine whether 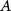 causes 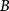 or 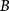 causes 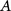.)

As has been addressed earlier, the reason for using MIME among the many other measures is that we have found it to be the most reliable and useful measure for our experimental data analysis. To verify the directionality of MIME in cross-brain analysis, two reading experiments (See subsection Causality verification of MIME) were analyzed, where MIME was found to be able to identify the correct direction of cross-brain interaction from the reader to the listener. We are convinced from this analysis that MIME does not generate false causalities and is reliable for the analysis of music experiments. Here we especially use the MIME causalities to construct networks that allow us to investigate the *differences* between experimental conditions.

## Results

We have used the causality measure MIME to analyze EEG data from music experiments, from these results we construct both intra-brain and cross-brain neural networks connecting large-brain regions of the musicians and listeners.

### Intra-brain neural information flow

In our analysis, each brain is considered as a neural network composed of large cortical brain regions connected by the neural information flow. A link is drawn in the intra-brain neural networks if the causality value is positive and significant according to a significance thresholding test (i.e. surpass the significance threshold, for details see the subsection Dependence on Thresholding), this way residual information flow [17] is avoided. We use time averages of the MIME causalities to determine the links between brain regions.

**Figure 1:**
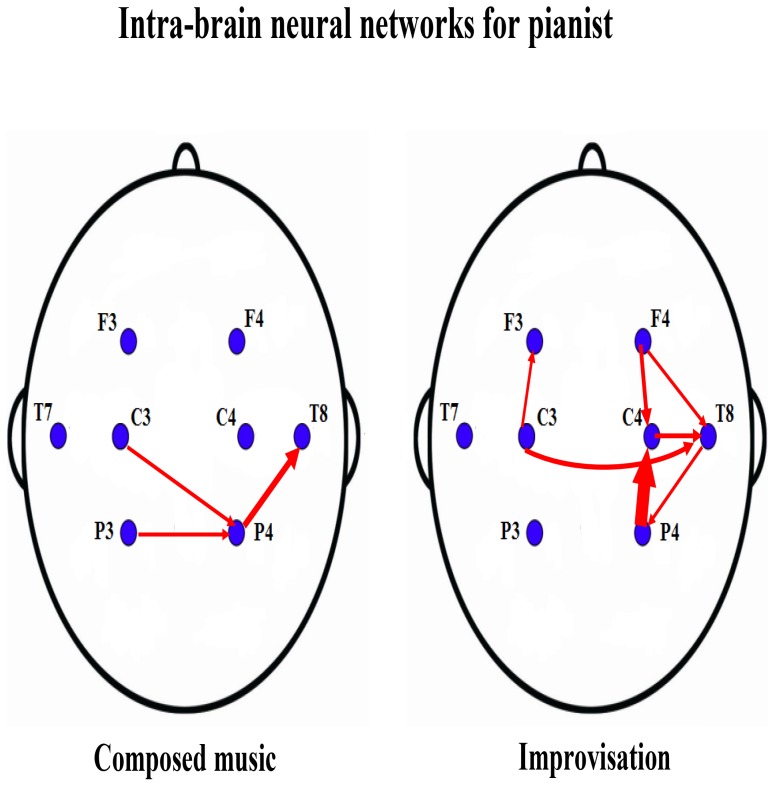
Pianist's intra-brain neural networks for the first experiment. The two panels show the pianist's intra-brain neural networks separately for composed music (left) and improvisation (right). The large brain regions are labeled by the 8 electrodes: F3, F4, C3, C4, T7, T8, P3, P4. The red links indicate the direction of neural information flow between large brain regions, where the thickness of the links represent the magnitudes of the causalities.

In the first experiment, the intra-brain neural networks for the pianist and the listener are shown in Figures 1 (pianist) and 2 (listener), respectively. The significance thresholds are taken as 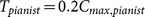 and 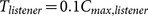 for the pianist and the listener, respectively. The 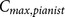 and the 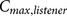 are the maximum causality values (averaged over time windows) for the pianist and the listener, respectively. The specific choice of the values 0.2 and 0.1 were found to produce the most clear difference between experimental conditions. Robustness of the results with respect to the choice of these parameters are discussed in subsection Dependence on Thresholding.

**Figure 2:**
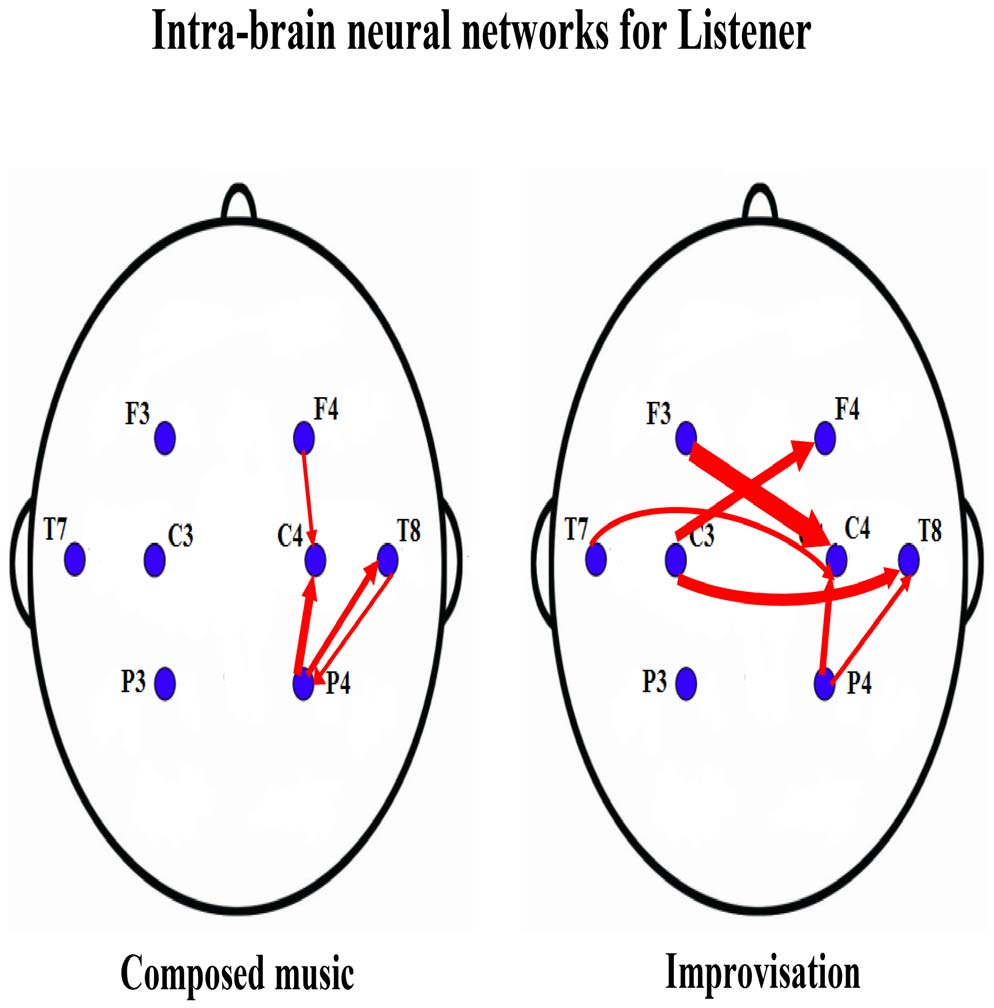
Listener's intra-brain neural networks for the first experiment. The two panels show the listener's intra-brain neural networks separately for composed music (left) and improvisation (right). The large brain regions are labeled by the 8 electrodes: F3, F4, C3, C4, T7, T8, P3, P4. The red links indicate the direction of neural information flow between large brain regions, where the thickness of the links represents the magnitudes of the causalities.

The intra-brain neural information flow networks differ between composed music and improvisation. For the pianist (Figure 1), the information flow is confined to the back of the brain during composed music, whereas during improvisation the flow expands to the entire brain. A similarly expansion was observed for the listener (Figure 2), although in this case the expansion is from the right part of brain to the entire brain when comparing composed music to improvised music.

**Figure 3:**
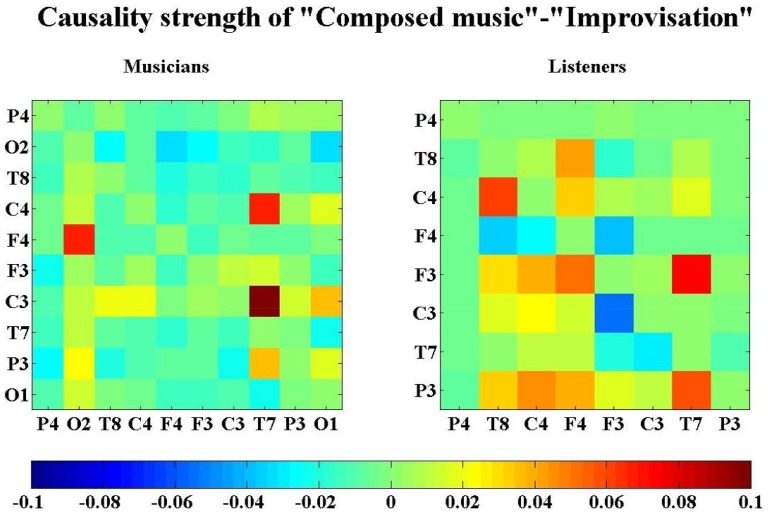
Color-map of the contrast causality matrix between composed music and improvisation. In this figure, the two 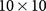 lattice plot indicates the contrast causality matrices between composed music and improvisation separately for musicians (left) and listeners (right). The direction of information flow is from the row channel to the column channel for each lattice. The color of the lattice indicates the strength of the causality contrasts, of difference, between composed music and improvisation, which is scaled between 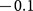 and 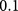. The correspondence between the color and the causality strength is shown in the color-bar.

In the second experiment, an extra pair of conditions: strict mode and “let-go” mode, was added to the experiment. To compare the differences between experimental conditions, we study the contrasts, computed as the difference, between the MIME causalities for the pairwise conditions, e.g. composed music versus improvisation and between “let-go” and strict mode. The contrast causality values were averaged over time windows separately for the musicians and the listeners. Again we used a significance thresholding test to decide the significance of the difference between the causalities. For each pair of conditions, e.g. the composed music vs improvisation (Figure 3), we define a radius 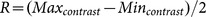 as half of the difference between the global maximum and the global minimum contrasts causality values, one radius for the musicians and one for the listeners. The significance threshold was defined as half of the radius 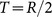, the contrast values outside the interval 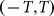 were deemed significant, otherwise insignificant. This definition of threshold is empirically reasonable, because a lower threshold will lead to a sharp increase in the number of detected information flow, while a higher threshold will prevent reasonable direction of information flow to be registered. For instance, the 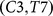 lattice site in the left panel (musicians) of Figure 3, which has the maximum contrast values 0.1, indicates significant information flow from 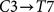, i.e. from the left central region to the left temporal region. For more details on the dependence on the specific value of the threshold see subsection Dependence on Thresholding.

**Figure 4:**
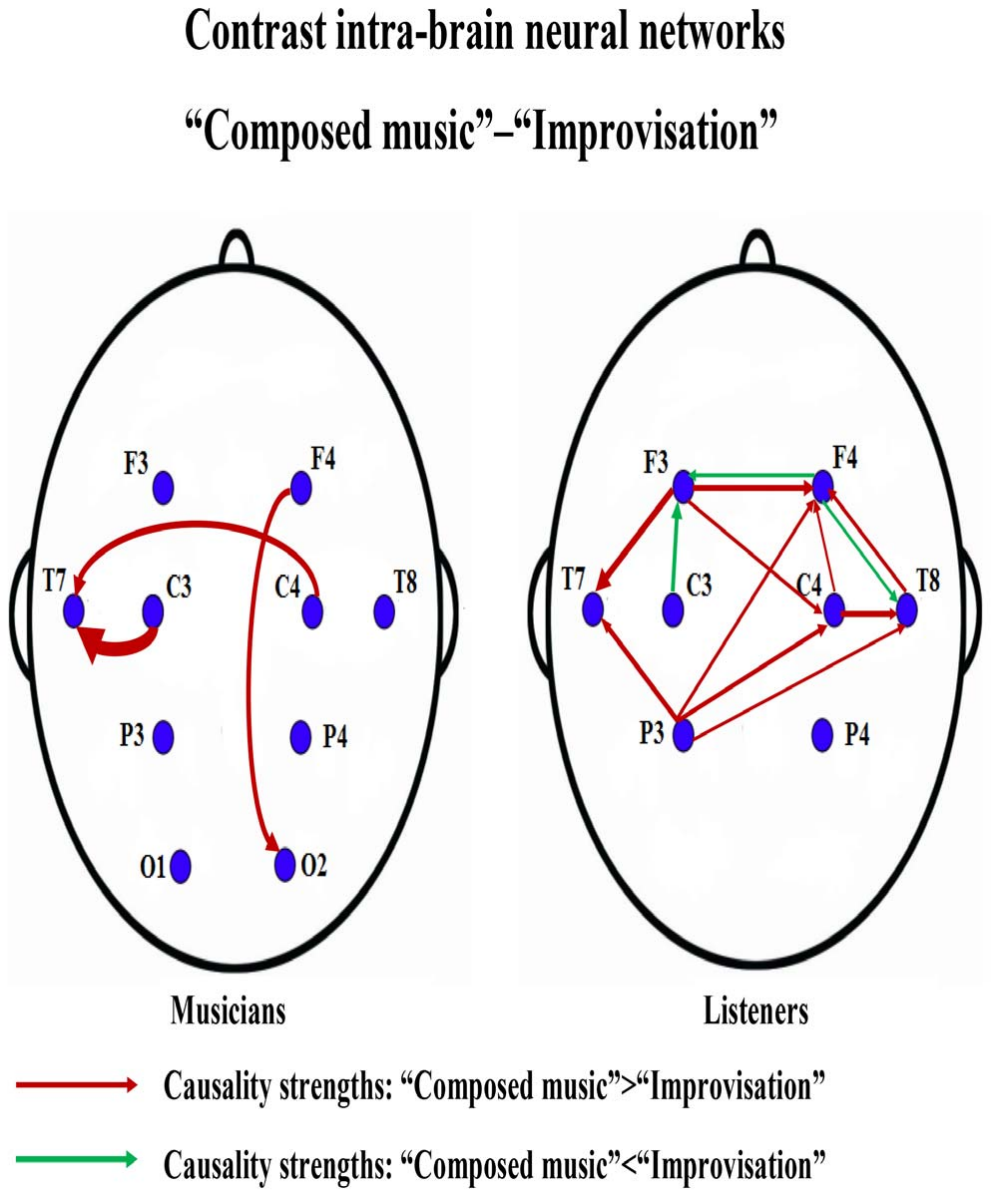
The contrast intra-brain neural networks between composed music and improvisation for the second experiment. The contrast neural networks were drawn from Figure 3. The causality contrasts were obtained by taking the differences of the MIME causalities between composed music and improvisation. A link is drawn in this network if the causality contrast is significant according to a thresholding test. The red links indicate the information flow that are significantly stronger (causality values) in composed music than in improvisation, while the green links indicate the information flow that are significantly stronger in improvisation than in composed music.

When composed music is compared to improvisation, we find that the composed music has overall stronger intra-brain causalities than the improvised, which is seen as more links (red) for “composed music > improvisation” than the links (green) for “composed music < improvisation” in the contrast intra-brain neural networks (Figure 4). This result does not contradict the observed expansion of neural information flow when composed music is changed to improvisation, this only indicates a difference in the strength of the causality values between the two conditions and corresponds to stronger values for the composed music.

For musicians (the left panel in Figure 4) the significant information flow of composed music are from both the left and right central regions to the left temporal region ([C3,C4]
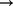
T7) and from the right frontal region to the right occipital region (F4
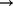
O2). For listeners (the right panel in Figure 4), information flow that are significant in composed music are from the left frontal and left parietal regions break into two branches, one is to the left temporal region ([F3,P3]
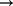
T7), the other is to the right frontal region via the right central ([F3,P3]
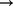
F4, or [F3, P3]
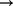
C4
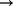
F4) and right temporal regions (P3
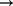
T8
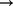
F4, or [F3, P3]
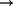
C4
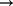
T8
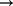
F4). The left frontal (F3) and left parietal (P3) regions act as the main sources of information flow, while the left temporal (T7) and right frontal (F4) regions are the main sinks, and the right central (C4) and right temporal (T8) regions serve as transit hubs. The listeners also have significant information flow during improvisation (green links in the right panel of Figure 4): from the right frontal region to the left frontal (F4
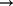
F3) and right temporal regions (F4
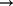
T8) and from the left central to the left frontal region (C3
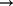
F3). The information flow that are significant during improvisation (red links) have directions opposite those found during composed music (green links). The more red links than green links in the figure of the contrast intra-brain neural networks are not in contradiction to the expansion in the distributions of information flow, when composed music is changed to improvisation. The dominance of red links only implies that those directions have stronger causality values during composed music than during improvisation. In other words, this analysis highlights the difference in causality values between the different music types. Namely, when the flow occurs with significant different causal weights for different mode of performance, it will be detected in Figure 4, while if the information flow occurs with more or less comparable and significant causality values for the two conditions no contrast will be registered in this figure. In the subsection Dependence on Thresholding, we explain that the observed contrast structure doesn't depend in any important way on the choice of thresholds.

**Figure 5:**
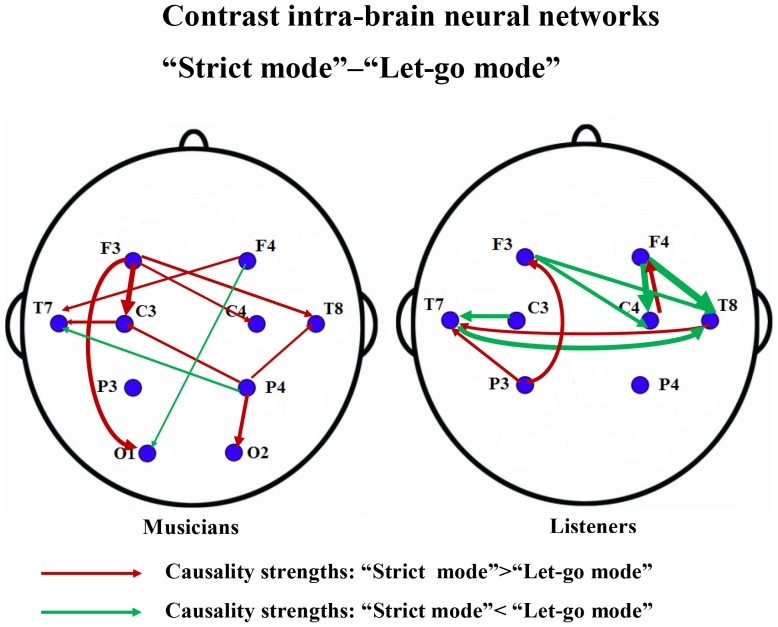
The contrast intra-brain neural networks between strict mode and “let-go” mode for the second experiment. The contrast neural networks were drawn from Figure 3. The causality contrasts were obtained by taking the differences of the MIME causalities between strict mode and “let-go” mode. A link is drawn in this network if the causality contrast is significant according to a thresholding test. The red links indicate the information flow that are significantly stronger (causality values) in strict mode than in “let-go” mode, while the green links indicate the information flow that are significantly stronger in “let-go” mode than in strict mode.

The network structures are more complicated for strict mode and “let-go” mode (Figure 5). For musicians (left panel) information flow that are stronger in strict mode (i.e. “strict
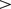
let-go”) are from the left frontal region to the left and right central regions (F3
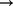
C3, C4) and to the left occipital (F3
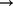
O1) and the right temporal (F3
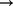
T8) regions, from the right frontal and left central regions to the left temporal region (F4, C3
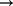
T7) and from the right parietal region to the left central (P4
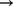
C3), right occipital (P4
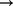
O2) and right temporal (P4
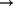
T8) regions. Here we see that the left frontal region (F3) and the right parietal (P4) region are key to musicians playing in strict mode (“strict
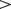
let-go”). However, in the same intra-brain neural network for musicians, information flow that are stronger in “let-go” (i.e. “let-go
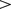
strict”) mode are from the right frontal region to left occipital region (F4
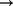
O1) and from the right parietal region to left temporal region (P4
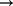
T7). For listeners, there is also a clear difference in the distribution of neural information flow. The information flow stronger in strict mode (i.e. “strict mode
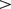
let-go mode”) are from the left parietal to the left frontal (P3
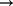
F3) and left temporal regions (P3
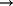
T7), from the right temporal region to the left temporal region (T8
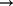
T7) and from the right central region to the right frontal region (C4
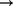
F4), whereas information flow stronger in “let-go” mode (i.e “strict mode < let-go mode”) are from the left and right frontal regions to the right central (F3,F4
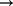
C4) and right temporal regions (F3,F4
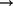
T8) and from the left central region via the left temporal region to the right central region (C3
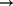
T7
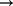
C4). In strict mode flow tend to be from the back to the front of the brain, whilst “let-go” mode tend to exhibit the inverse direction from the front to the back of the brain. We explain in the subsection Dependence on Thresholding that the observed contrast structure doesn't change when we change the thresholds values by small amounts.

Since the intra-brain analysis studied the difference between different experimental conditions, we do not have enough statistics to discuss reliably person specific instantaneous flow patterns, but have concentrated on average trends of information flow as well as the sink and source activities of large brain regions. These results are obtained by averaging over time windows and over experimental conditions.

### Degree centrality analysis

To identify the difference between experimental conditions in terms of the importance of large brain regions, we carried out an analysis of the degree centrality of the intra-brain neural networks. Since the intra-brain neural networks are directed we count the number of in-going and out-going links to a node separately and thereby calculate the in-degree and out-degree for each node (i.e. large brain region). The degree centralities were averaged over time windows and experimental conditions, results show that the musicians typically have opposite trends to the listeners when composed music is compared to improvised and the strict mode is compared to the “let-go” mode.

**Figure 6:**
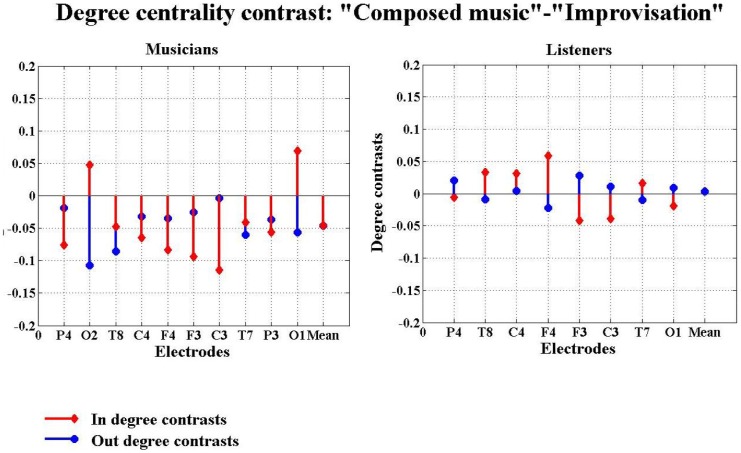
Degree centrality contrasts, or difference, between composed music and improvisation in the second experiment. In this figure, the red stems and the blue stems indicate the in and out degree centrality contrasts between composed music and improvisation, respectively. The horizontal axis has 9 channels represent the 8 electrodes: P4, T8, C4, F4, F3, C3, T7, P3 and the overall average over the 8 electrodes, while the vertical axis gives the magnitudes of the degree centrality contrasts between composed music and improvisation.

**Figure 7:**
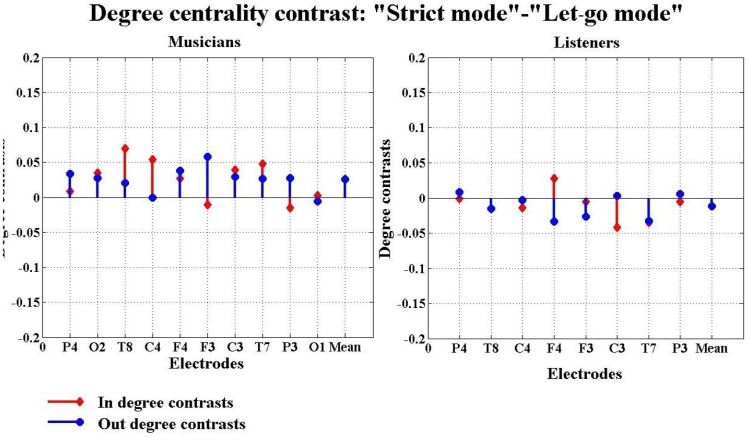
Degree centrality contrasts between strict mode and “let-go” mode in the second experiment. In this figure, the red stems and the blue stems indicate the in and out degree centrality contrasts between strict mode and “let-go” mode, respectively. The horizontal axis has 9 channels represent the 8 electrodes: P4, T8, C4, F4, F3, C3, T7, P3 and the overall average over the 8 electrodes, while the vertical axis gives the magnitudes of the degree centrality contrasts between strict mode and “let-go” mode.

The differences between the experimental conditions were compared by subtracting the degree centralities found under one condition from those found under another condition and thereby focus on the contrast between experimental conditions. In Figure 6, we show that musicians were found to have larger in- and out-degrees during improvisation than during composed music, while the listeners exhibit the opposite trend. When strict mode was compared with “let-go” mode (Figure 7), we find that musicians have larger in- and out-degrees in strict mode than in “let-go” mode, while listeners again exhibit the opposite results. In this analysis, a larger in- and out-degree indicates a larger amount of information flow in and out of the coresponding brain region and hence one would expect this to imply that the region is more functionally involved with the other regions in the network.

### Cross-brain networks

**Figure 8:**
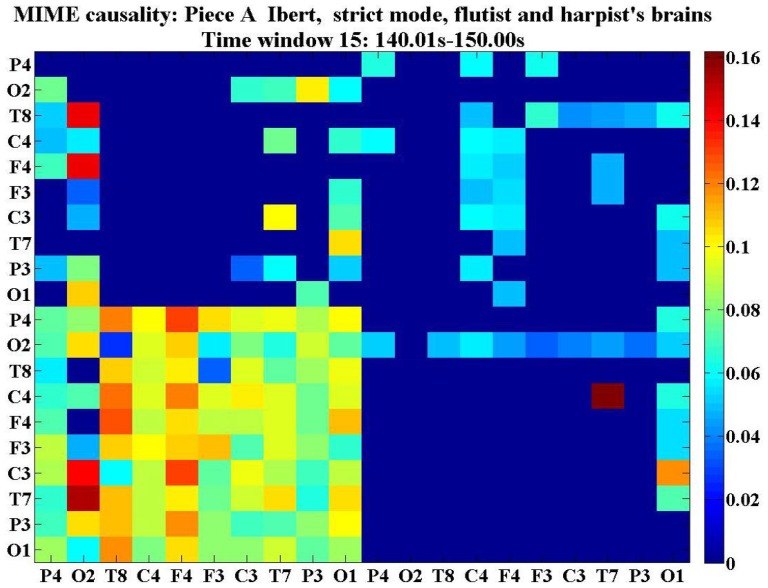
Color-map of cross-brain causality matrix between flutist and harpist in the second experiment. This graph shows a color-map (scaled between 0 and 1) of the cross-brain causality matrix for the flutist and harpist at the time window 15 (140.01s-150.00s) during the performance of piece A: Ibert (strict mode). The two 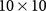 diagonal blocks indicate the intra-brain causalities for the flutist (upper-left) and the harpist (lower-right), respectively, while the two 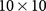 off-diagonal blocks indicate the cross-brain causalities for flutist
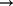
harpist (upper-right) and for harpist
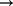
flutist (lower-left). The correspondence between the color and the causality values is shown in the color-bar.

**Figure 9:**
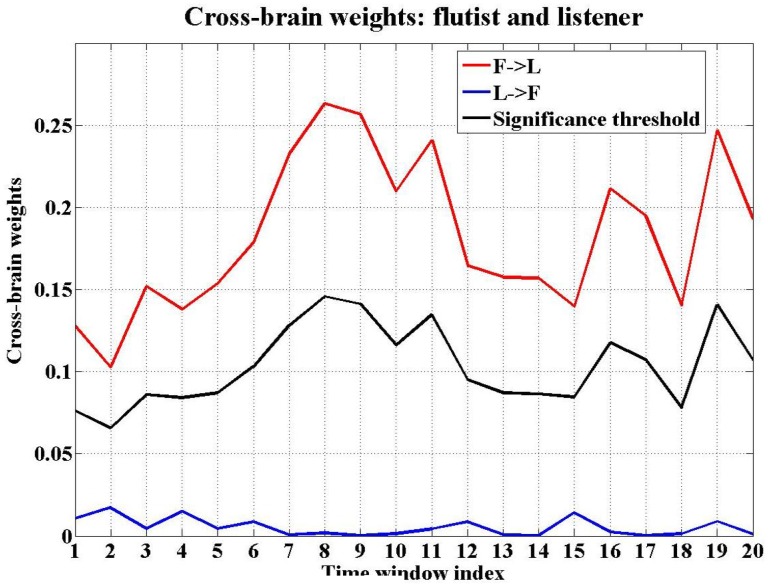
The cross-brain weights between flutist and listener in the second experiment. This figure plots the cross-brain causalities between flutist and listener against time windows for piece A: Ibert, strict mode. The red curve indicates flutist
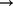
listener, the blue curve represents listener
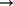
flutist, while the black curve is the significance threshold.

P. Vuust reported in [28] and [29] a study of jazz performances, where the jazz musicians were found to communicate with each other by modulating their individual rhythm during ensemble performances. In our experiments, we study both the musicians and the listeners, and we try to investigate the way the musicians coordinate with each other in terms of information flow. In Figure 8, we show e.g. the information flow within and between the brains of the flutist and the harpist. We also investigate how the musicians interact with the listeners during the music performances. In our study, to analyze the pattern of coordination, we monitor the average cross-brain causalities which results in a single nonnegative real number for each direction (i.e. from one brain to another). A cross-brain link is drawn if the average causality value i.e. the cross-brain weight, is significantly higher in one direction than in the opposite direction. For instance, in the second experiment, the cross-brain weight was significantly higher for harpist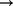 listener, but almost vanished for listener
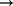
harpist, hence the cross-brain interaction was from the harpist to the listener during the music performances. We do not need to define a specific threshold, since the cross-brain weight is positive with high values in one direction and almost vanishing cross-brain weight in the opposite direction between each pair of brains. For instance, in Figure 9, the cross-brain weight for flutist
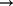
listener is clearly higher than the cross-brain weight for listener
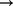
flutist, which implies a directed link from the flutist to the listener.

**Figure 10:**
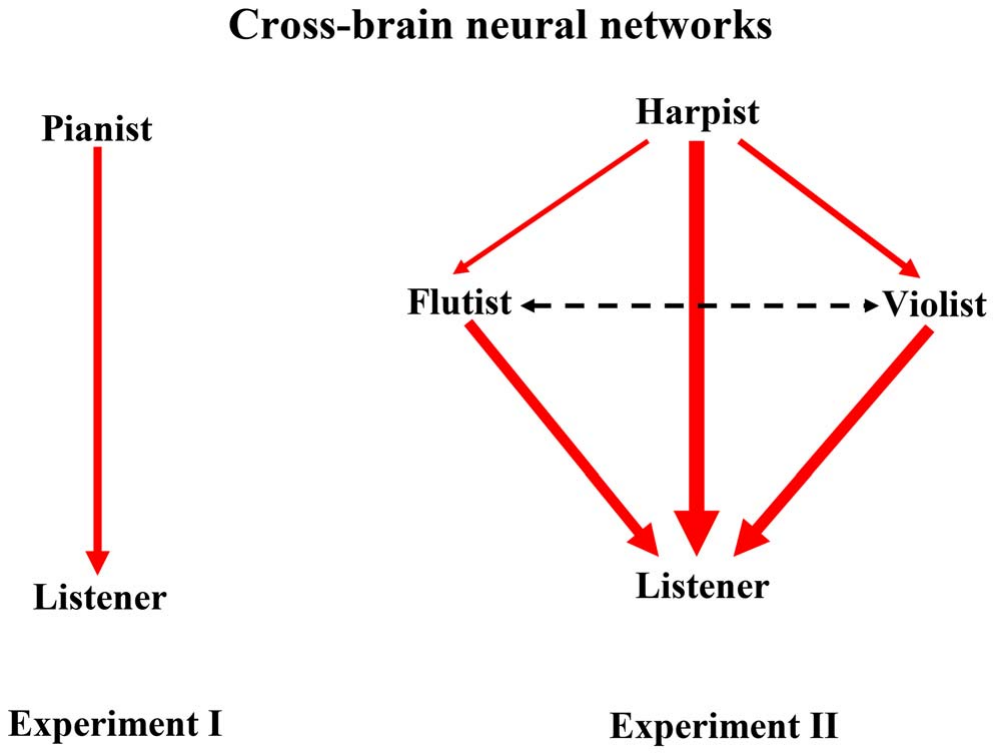
Cross-brain networks for the two music improvisation experiments. The left graph is for the first experiment, while the right graph is for the second experiment. The red links represent the direction of cross-brain information flow, while the thickness of the links is proportional to the strength of the cross-brain weights (i.e. the average cross-brain causalities).

In the first experiment (the left graph of Figure 10), the cross-brain interaction is from the pianist to the listener (average weights: 
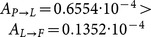
), while in the second experiment (the right graph of Figure 10), the cross-brain interactions are from the three musicians to the listener: [flutist, harpist, violinist]
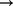
listener (average weights: 
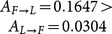
, 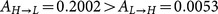 and 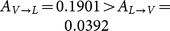) and from the harpist to the flutist and violinist: harpist
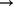
[flutist, violinist] (average weights: 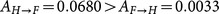 and 
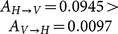
). The flutist ping-pongs with the violinist: flutist
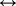
violinist (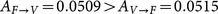), the average values are high in both directions, but the dominance of the cross-brain weights swaps between the two when the time window moves). This network structure is robust for all performances in the second experiment.

To verify the directionality of MIME in the detection of cross-brain interactions, we conducted two reading experiments. The reading experiments consist of a reader and a listener, where the reader read to the listener to establish a natural driver-responder system during the reading processes. Each reading experiment has two tests, where the reader and the listener swap their roles for the different tests. The EEG data of the reading experiments were analyzed by MIME to obtain the cross-brain information flow. The outcome is a causal direction pointing from the reader to the listener. We mention that in one test of each experiment the cross-brain weights for the flow between the reader and the listener were equivalent, but, importantly, both weights were insignificant, in which case no cross-brain interaction is detected. This is of course a limitation of the MIME analysis and shows that MIME may miss causal relations. On the other hand, the analysis of the reading experiment suggests that MIME is unlikely to produce causalities that do not exist. In other words, we believe that MIME is unlikely to produce false positives. For more details see subsection Causality verification of MIME.

## Discussion and Summary

In this paper, we constructed intra-brain and cross-brain networks for both musicians and listeners during music performances. The differences between the composed music and improvised music and between the strict mode and the “let-go” mode can be identified in terms of the direction of neural information flow, the number of in-going and out-going connections (i.e. the in-degree and out-degree centralities) between large brain regions, as well as the sink and source activities in the frontal, parietal and temporal regions. The latter are similar to the results obtained from the sLORETA on the same data set [10], [8].

In the intra-brain neural network analysis, the improvisation was found to trigger a more widely distributed network structure than the composed music did. When composed music is changed to improvisation, the distribution of intra-brain neural information flow expands from the back of the brain to the entire brain (for musicians), the frontal (attention and executive control) and central (motor cortex) regions become activated when musicians improvise. This may be because performing or listening to improvisations demands more widespread functional coordinations between large brain regions. When composed music is compared to improvisation, the intra-brain causality values are found to be greater in composed music than during improvisation, particularly for the listeners. We find that the neural information flow starts and terminates separately in left frontal and right frontal regions, the neural information flow reverses directions when composed music is changed to improvisation and strict mode is changed to “let-go” mode. These results agree with earlier studies [7] [22]: the frontal regions (a more general area that covers the dorsal prefrontal regions), especially the right frontal region, play an important role in free improvisation of melodies and rhythms, which is the key regions that distinguish the brain activities between composed music and improvisation and between strict mode and “let-go” mode. Moreover, the central regions tend to act as transit hubs for the neural information flow for *all* experimental conditions. This is in contrast to what we find for the temporal and parietal regions, which behave differently under different experimental conditions.

The identification of the importance of the frontal regions is similar to the findings of a previous fMRI study of pianist improvisation [2], where the dorsal prefrontal cortex (part of the frontal regions) and rostral premotor regions (located within the frontal regions) were found to be involved in the free-response selection. This study shows an activation of the cortical association areas, especially the prefrontal cortex, during divergent thinking, where the right prefrontal cortex appears to be particularly involved. The high level of involvement of the frontal and central regions and the source activity of the right frontal region during improvisation also agree with the cortical source analysis (sLORETA) on the EEG data we have studied in this paper (see [10]). Dolan et al. found that a clear increase in the activation of the frontal region, acting as the EEG point sources of the brain activities, when composed music was changed to improvisation [10]. A similar studies on cortical regions of music improvisation used fMRI on a improvising pianist [4]. The study found the dorsal prefrontal and rostral cingulate regions to play a key role in melodic and rhythmic improvisation [4].

In the study of intra-brain neural networks we used the degree centrality to analyze the level of connections between large brain regions. Since the intra-brain neural networks are directed and the degree centrality measure (i.e. the number of links connected to the nodes) is very simple to use and is a very suitable centrality measure for directed networks. In this analysis musicians were found to have opposite trends to the listeners. The musicians tend to have overall larger (in and out) degree centralities in improvisation than in composed music, which may be because the improvisation demands more intra-brain communication for the musicians to be able to instantaneously create melodies and rhythms. They also have larger degree centralities in strict mode than in “let-go” mode, which may be because musicians need more brain attention to perform in strict mode. In contrast, the listeners have larger degree centralities in composed music than in improvisation, which may be because the listeners found the music performed according to a score to be more familiar than the instantaneous creation of music during improvisation. The listeners also have larger degree centralities in “let-go” mode than in strict mode. It is interesting to mention that a questionnaire answered by the listeners showed that music performed with free emotional expression, i.e. the “let-go” mode, is considered more beautiful than the mechanical rendition of music, i.e. the strict mode, see [10] for details.

The cross-brain network structure provides a sensible view of the pattern of coordination between musicians and interactions between musicians and listeners, either during solo, or ensembles, performances. In the cross-brain networks, the musicians are pointing to the listeners, which of course seems to confirm the fact that the musicians are communicating *to* the listeners during the music performances. The harpist was frequently found to lead the flutist and the violinist, this may be because the harp provides the chord structure, which is then responded to by the flutist and the violinist during the trio's improvisation.

We want to point out a limitation and a strength of our study. Our EEG recording has only 8 or 10 electrodes, which are quite few compared to other studies. This enables us to analyze only general brain activities. However, since we do not focus on specific task-related brain activities, this experimental set-up is sufficient for us to be able to distinguish the brain activities from different cortical brain regions and at the same time the low number of electrodes minimize the discomfort to the participants during the experiments. Nevertheless, the results of our study imply that the neural differences in the brain of the subjects (e.g. the musicians and the listeners) under different experimental conditions (e.g. composed music and improvisation) can be detected by the network analysis generated from the MIME causality measures. This analysis provides a potential tool to study the intra-brain and cross-brain information flow and therefore is a very promising tool for the analysis of group behavior in other situations similar to ensemble performances of music. The method of analysis can potentially be applied to financial and more general neuroscience data sets.

## Methods

The experiment on musicians has already been published in [10] and received ethical approval from Guildhall School of Music and Drama.

The reading experiment was performed on employers from Brainmarker. Before agreeing to participate, the participants were informed in detail about the experiment and signed the informed consent form; although they were allowed to withdraw at any moment. The reading experiment simply consists of the same kind of check-of-method as carried out routinely by, and on, Brainmaker's employees, hence no institutional approval was obtained. We have obtained confirmation from the Head of the Imperial College Mathematics Department that prior approval was not required.

We use causality measures to analyze the intra-brain connectivities between large brain regions and cross-brain interactions between musicians and listeners. We have tried three frequently used causality measures, namely the partial directed coherence (PDC [25] [26]), transfer entropy (TE [24]) and conditional mutual information from mixed embedding (MIME [27]), in order to compare the efficiency and practicality in EEG analysis. From the analysis, we found that PDC gives an unrealistic large number of cross-brain causalities from listener to the pianist in the first experiment although listener and pianist were facing away from each other. TE has poor directionality as it gives similar strength for causalities between pairs of links with opposite directions. Only MIME presents clear directionality and robust results with larger average causalities from musicians to listeners than from listeners to musicians. Therefore, we use MIME as our core causality measure for the EEG analysis.

The MIME software package developed by I. Vlachos and D. Kugiumtzis, et al. [27] was used to calculate the causalities between EEG data channels. MIME is a time domain bivariate method, used to analyze nonlinear indirect information flow. It uses a progressive scheme to select mixed embedding vectors that maximizes the conditional mutual information rate between future and past embedding vectors [27]. For a K-dimensional stationary vector process 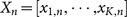, the causality from 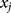 to 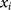 is calculated by defining a future vector 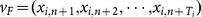 containing the future of the driven variable (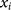), a uniform state-space embedding vector 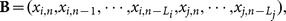
consists of the lagged values from both driving (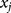) and driven (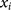) variables and an empty vector 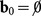 as an initial selected non-uniform state-space embedding vector, 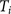 is the time horizon (prediction step) of 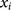 and 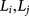 are the maximum time lags for 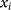 and 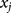, respectively. In each iterative cycle 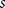, the progressive scheme seeks element in 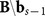 that satisfies the maximum criterion 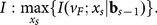
(1)which element will be add to 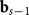 to form a new selected vector 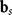. The progressive scheme stops at an 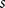-th iterative circle and uses 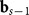 as the final embedding vector if the stopping criterion 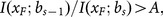
(2)is satisfied. Here, 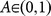 is a threshold close to 1. I. Vlachos and D. Kugiumtzis, et al. [27] found empirically that 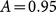 (default in MIME software) gives the best causality detection.

When the progressive scheme terminates, MIME measures the causal effect from 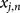 to 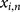 (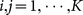, 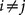) by evaluating the ratio between the conditional mutual information rates 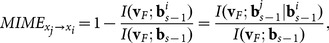
(3)where 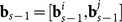 is the final selected non-uniform state-space embedding vector when the progressive scheme terminates where 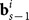 and 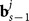 are the 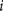 th and 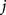 th components of 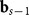, respectively.

Here, MIME was applied on the standardized EEG voltages. MIME is an information based measure entirely determined by the probability distributions of the signals and therefore independent of the amplitude of the measured signal. Hence no normalisation is necessary. To analyze cross-brain information flow, synchronized EEG data measured from each combination of two different brains was put together to form an augmented data matrix, e.g. the EEG data of the pianist and the listener. These augmented data matrices were analyzed by moving time windows with window size 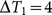
s (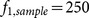
Hz) for the first experiment and 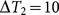
s (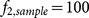
Hz) for the second experiment. These time windowed data files were used as input to the MIME software.

The MIME software outputs sequences of causality matrices, which contain both intra-brain and cross-brain causalities. These matrices are of size 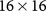 (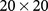) for the first (second) experiment, which consists of two 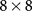 (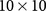) diagonal sub-matrices for intra-brain causalities and two 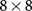 (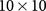) off-diagonal sub-matrices for cross-brain causalities. The diagonal sub-matrices (intra-brain) were averaged over time windows to construct intra-brain neural networks. The intra-brain causality matrices were also discretized into binary matrices, which after matrix transposition become the directed adjacency matrix for the intra-brain neural networks, the directed adjacency matrices were then used to compute the degree centralities. The off-diagonal sub-matrices (cross-brain) were used to construct cross-brain networks by taking averages of the cross-brain causalities over electrodes and comparing the magnitudes of the causality averages with the opposite cross-brain direction. A cross-brain link is drawn from one brain to another, if the average cross-brain causality is significantly larger from one brain to the other than measured in the opposite direction. If the average values are equivalent in both directions, the cross-brain causality cancel each other and one will not draw a link between this pair of brains.

A strength of MIME is that it doesn't rely on any computationally costly significance tests. This is due to the stopping criterion and the progressive scheme. However, we do have to use significant thresholding tests on the MIME causalities in order to identify the important differences between experimental conditions and filter out any residual flow arising from numerical inaccuracies [17]. We made use of different values for the thresholds for the different types of analysis reported. In all cases we chose the threshold with the aim to best identify neural difference between experimental conditions. It is important to point out that small changes to the value of the thresholds do not have any serious influence on the results. Details of the investigation of the significance threshold are presented in subsection: Dependence on Thresholding.

### Causality verification of MIME

We use MIME for our EEG analysis because it is found to be more reliable than TE [24] and PDC [25], [26]. Several simulation tests find that MIME is able to detect correctly the causality structure [27]. Moreover, reasonable directional interdependencies are reported for experimental time series such as EEG for epilepsy patient [27]. To our knowledge, no paper has used MIME to study music improvisation studies yet. To verify the directionality of MIME, we designed two reading experiments with the aim to check the cross-brain directional inference of MIME.

The reading experiments include one reader and one listener, both of which are healthy normal people. The reader is to read a short story to the listener, while the listener is to listen to the story carefully and try to imagine the scene described by the story. When the first story is finished the reader and the listener swap their roles after a short break, to repeat the reading process on another story. The stories were new to both the reader and the listener. The reader and listener did not face each other during the tests in order to avoid visual influences. Synchronized EEG data was measured from the reader and the listener on 10 electrodes (P4, O2, T8, C4, F4, F3, C3, T7, P3, O1) during the reading processes with 100Hz sampling frequency. The whole experiment was repeated once on another pair of healthy normal subjects to avoid fortuity.

The MIME analysis (time window analysis, window size: 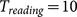
s) shows that the dominant cross-brain information flow is from the reader to the listener. In the first reading experiment, the average (cross-brain) causalities are 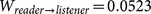 and 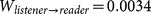 in one test, while 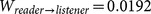 and 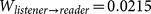 in the other test. In the second reading experiment, 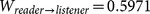 and 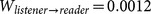 in one case, while 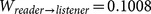 and 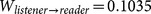 in the other test. For both experiments, a link can be drawn from the reader to the listener, rather than the opposite direction, because the overall causality average is significantly greater for reader
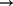
listener than for listener
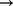
reader. This is according to a significance thresholding test with instantaneous threshold 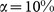 above the mean value between 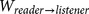 and 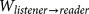 at each time window. For the cases with equivalent causality strength between the reader and the listener, non of the two causal directions surpass the significance threshold, in which case it was deemed that no significant causal influence occurred between the reader and the listener. Nevertheless, the overall average for the four tests (two experiments) gives dominant cross-brain causalities from the reader to the listener.

We have varied the parameters of MIME, e.g. the time horizon (prediction step) 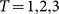 and the maximum embedding dimension (time lags) 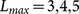 under restriction that 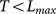 [27]. In spite of these variations the directional results were unchanged. This implies that the directionality of MIME doesn't depend strongly on the parameter choice. Our conclusion from the reading experiments is that MIME may fail to pickup up causal links (e.g. no significant causal influence between the reader and the listener), but it never predicts an unreliable causalities. This means, once MIME picks up a causal direction, one has good reason to believe in the directional results.

### Dependence on Thresholding

The network exhibiting the information theoretic causality flow between brain regions relies on defining thresholds. If the strength of the causal weight in a certain direction exceeds the threshold, a directed link is included in the network. We now study systematically for each of the extracted networks, how the resulting structure depends on the value of the chosen threshold.

### The First experiment

In Figures 1 and 2 different thresholds were used for the pianist (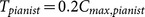) and for the listener (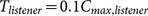). This choice of thresholds correspond to the most clear difference between experimental conditions. In order to access the stability of our results we altered the thresholds to intervals with boundaries 10% below and above the original thresholds, namely, 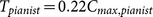 and 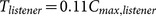 or 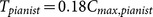 and 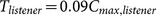. For this new set of parameters the intra-brain neural network structures for both the pianist and the listener remain unchanged. We also checked what happens when we changed the thresholds by 100% below and above the original thresholds. In this case changes do occur to the network structures but only involving the weaker links, the stronger links i.e. the most dominant links still remain unchanged. Thus the conclusion concerning the main differences between the different modes of performance remain unchanged

### The Second experiment

We again check how the 10% changes to the thresholds influence the network structures. See Figure 4 for the contrast intra-brain neural networks between composed and improvised music. When the significance thresholds increase by 10%, i.e. a new interval 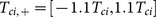 (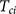 denotes the original threshold), the contrast network structures remain unchanged for both the musicians and the listeners. When the significance thresholds decrease by 10%, i.e. a new interval 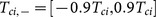, the contrast network structure is unchanged for the musicians, while for the listeners, all the old links remain, with new red link (causalities Composed music
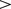
improvised music) F3
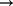
T8 and green links (causalities Composed music
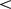
improvised music) F4
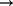
C4 and T7
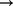
C3 added to the network. However, these small changes to the listeners' network, follow the same pattern as the old links do, i.e. the trends of the neural difference between the composed music and the improvisation remain unchanged.

For the contrast between the strict mode and the “let-go” mode in Figure 5, again the networks for both musicians and listeners stay unchanged when the significance threshold increases by 10%, i.e. 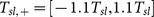. However, when the significance threshold decreases by 10%, new red links (causalities Strict mode
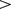
“let-go” mode) T8
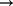
O2 and P3
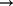
[C4,T7] and new green links C4
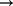
T7 and C3
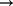
F3 are added to the musicians contrast network, whereas new red links T8
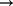
F4 and P3
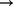
F3 and new green links F4
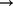
[T8,C4] are added to the listeners contrast networks. Nevertheless, the new links follow the same pattern as the original links do and hence no essential change occurred to the observed neural differences between the strict mode and the “let-go” mode.

From this analysis we conclude that small changes to the significance thresholds do not have any essential influence on the neural network structures, which implies that the observed neural differences between experimental conditions are robust.

As to the cross-brain networks, the significant cross-brain links are drawn in the dominant directions (i.e. the direction with stronger weight between a pair of opposite directions). Because the cross-brain weights are significantly positive in one direction and almost vanished in the opposite direction, the cross-brain networks are robust and do not necessitate the use of any significance thresholds.

### Selection of causality measures

There are a number of reasons for us to use MIME in our EEG analysis. Firstly, we have compared our EEG analysis using three popular causality measures: MIME [27], PDC [25] [26] and TE [24], in which MIME produces the most reliable results among the three measures. PDC is a linear method which relies crucially on linear autoregressive models. For real EEG analysis, PDC presents a large number of, presumably false, causalities from the listeners to the musicians. The PDC causality flow from listeners to musicians was found even when the two groups couldn't watch each other and since only the musicians made any sound we would expect causality to entirely flow from musicians to listeners.

TE is a nonlinear method, which is supposed to work better than PDC in nonlinear time series analysis. However, due to computational restrictions on embedding dimensions, TE cannot use large enough embedding dimensions and is for this reason unable to produce satisfactory directional results. The TE's analysis generates similar causalities between every pair of brains, so no cross-brain network structure is detected. A small increment in the embedding dimension will cause a dramatic increase in the computation time.

Both the linearity and computational short comings of the PDC and TE were overcome by MIME, which can produce reliable causality results efficiently [27]. As has been tested on various data, MIME presents all correct directional results for model data and reasonable causalities for experimental time series [27]. Furthermore, we also did tests on a natural driving-driven respond system, namely the reading experiments to test the reliability of MIME in cross-brain analysis (See section Causality verification of MIME), from which analysis we concluded that MIME does not present false or unreasonable causalities when analyzing directed interactions between experimental time series.
